# Validating deep learning inference during chest X-ray classification for COVID-19 screening

**DOI:** 10.1038/s41598-021-95561-y

**Published:** 2021-08-09

**Authors:** Robbie Sadre, Baskaran Sundaram, Sharmila Majumdar, Daniela Ushizima

**Affiliations:** 1 Computational Research Division, Lawrence Berkeley National Laboratory, Berkeley, CA 94720 USA; 2 Department of Radiology, Thomas Jefferson University Hospital, Philadelphia, PA 19107 USA; 3 Institute for Computational Health Sciences, University of California, San Francisco, CA 94117 USA; 4 Berkeley Institute for Data Science, University of California, Berkeley, CA 94720 USA

**Keywords:** Software, Pathology

## Abstract

The new coronavirus unleashed a worldwide pandemic in early 2020, and a fatality rate several times that of the flu. As the number of infections soared, and capabilities for testing lagged behind, chest X-ray (CXR) imaging became more relevant in the early diagnosis and treatment planning for patients with suspected or confirmed COVID-19 infection. In a few weeks, proposed new methods for lung screening using deep learning rapidly appeared, while quality assurance discussions lagged behind. This paper proposes a set of protocols to validate deep learning algorithms, including our ROI Hide-and-Seek protocol, which emphasizes or hides key regions of interest from CXR data. Our protocol allows assessing the classification performance for anomaly detection and its correlation to radiological signatures, an important issue overlooked in several deep learning approaches proposed so far. By running a set of systematic tests over CXR representations using public image datasets, we demonstrate the weaknesses of current techniques and offer perspectives on the advantages and limitations of automated radiography analysis when using heterogeneous data sources.

## Introduction

The novel coronavirus has killed more than half a million Americans out of the two and a half million deaths worldwide as of March 2021, according to the Centers for Disease Control and Prevention (CDC). These RNA viruses use their protein-based spikes to break into the human cells using human ACE2 receptors^1^, often leading to severe acute respiratory syndrome coronavirus 2 (SARS-CoV-2), characterized by the development of pneumonia together with other symptoms such as fever, cough, and loss of sense of smell^2^. In the most severe cases, this viral infection unleashes an aggressive auto-inflammatory response known as the cytokine storm that causes the body to attack itself, sometimes leading to lifelong organ damage.

What was initially thought to be a respiratory-virus-only soon became known to manifest itself in several parts of the body, with a long list of symptoms from arrhythmia, heart attacks, blood clots to damaged liver and kidneys^3^, rashes and more. Nonetheless, respiratory abnormalities continue to be the most prominent sign of COVID-19. In terms of diagnosis, specificity for COVID-19 using thoracic radiography is highly controversial^4^ and the suitability of radiography for frontline prescreening continues to be disputed. For example, several radiology organizations, such as the American College of Radiography recommends against performing diagnoses using clinical radiography for the identification of COVID-19^4–6^. Nonetheless, a few researchers support the idea that lung scans examination could be used as a primary tool for screening in epidemic areas^7,8^, and of invaluable use worldwide as important information for diagnoses^9–11^, and particularly for the management of respiratory tract infections^6^.

**Figure 1:**
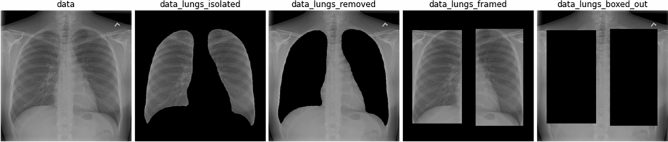
From raw chest X-ray to modified image version for testing deep learning inference: proposed representations to inspect lung classification accuracy in the ROI Hide-and-Seek protocol.

Due to the limitations of tests such as polymerase chain reaction with reverse transcription (RT-PCR) early on in the pandemic, and the urgency for developing new measures to control the COVID-19 spread, a plethora of machine learning methods using lung scans have been proposed in the past year^12–15^. There have also been numerous efforts in federating lung scans into a common repository, the largest one being COVIDx, an open access benchmark dataset comprising thousands of chest X-ray (CXR) images^13^ and the largest number of publicly available COVID-19 positive cases to date used for classification. These recent contributions discussed the advantages of leveraging CXR imaging for COVID-19 screening, but many questions remain unanswered, such as:

- If we change CXR inputs to focus on the lung region before classification, do CNN-based methods perform better?
- Does the removal of key CXR image portions affect the diagnostic accuracy, sensitivity and positive predictive values (PPV)?
- Could classification methods be identifying data sources as opposed to key features from the lungs? (Fig. 1)

In order to answer these questions, this paper presents a systematic set of protocols necessary to validate deep learning models during decision-making based on CXR, pointing out to the dangers of those approaches that can be oblivious to relevant information from the lungs. In addition, we investigate the role that the lung segmentation might play in the CXR classification process, particularly when including both data sources with previously known respiratory infection cases^16^ and COVID-19 specific imaging^17^. The main contributions of this investigation are the development of:

- Protocols to measure ML models when using heterogeneous data sources, particularly with an number of patient cases;
- Strategies to verify that the visual features detected by the ML models are specifically recording the locations of lung abnormalities as opposed to bright artifacts, such as medical equipment or hard tissue;
- Algorithms to monitor the position of a feature used in the CXR image processing task, and to assess the correlation to critical factors associated with COVID-19.

**Figure 2:**
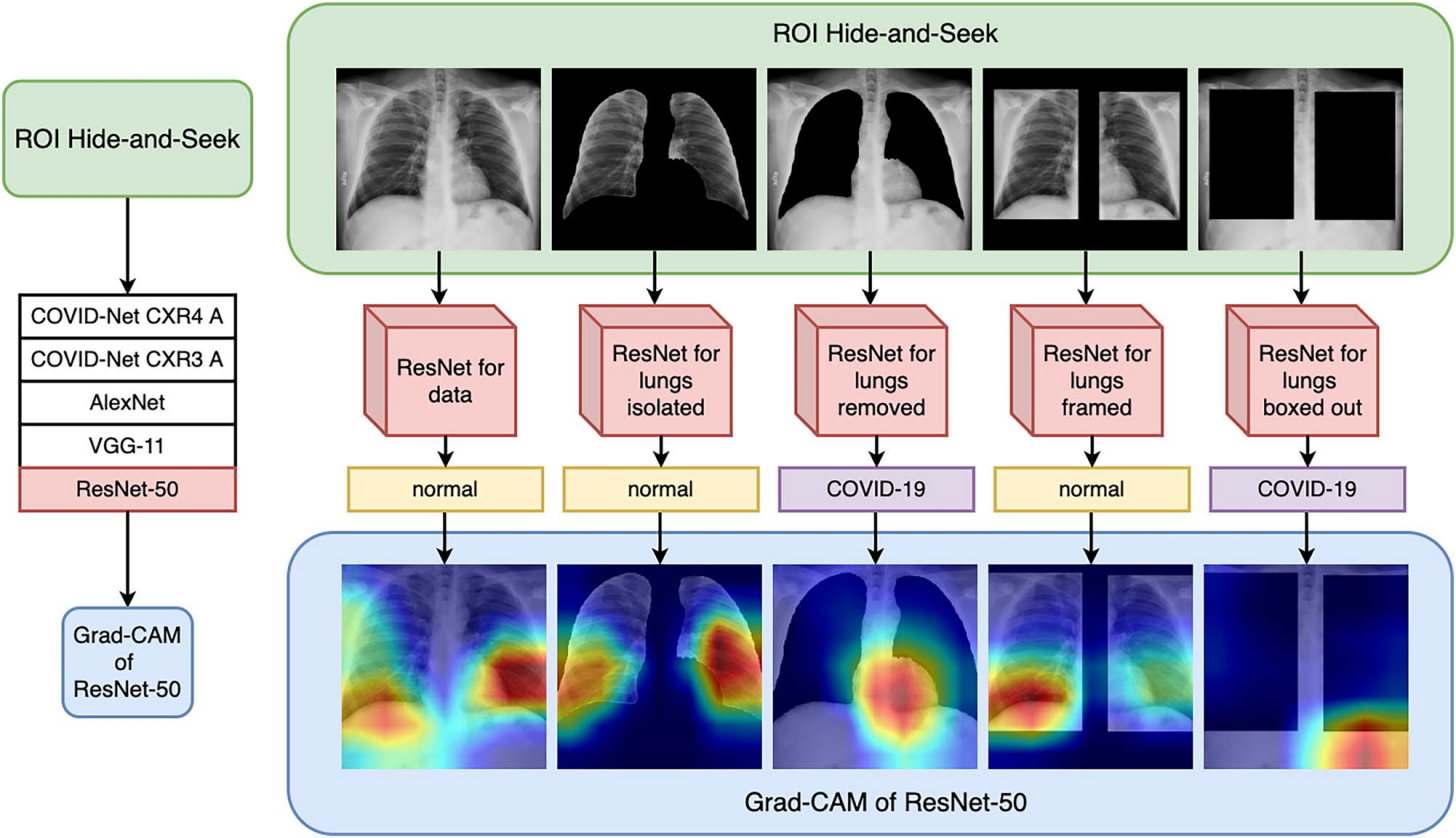
From raw chest X-ray to ROI Hide-and-Seek for validating deep learning inference.

The outline of this paper is as follows. We first discuss state-of-the-art research to show potential gaps when using neural networks to process radiography images. We then introduce protocols and strategies for assessing deep learning models for segmentation and classification, applied to the open access benchmark dataset COVIDx. The following section describes the experimental results when including or excluding lung segmentation, a method we call “Region of Interest (ROI) Hide-and-Seek”; which uses a U-net to detect the lungs as prior information to create 5 different image representations of the CXR data. Each set of CXR image representations is used to train and test each of the 5 different deep learning architectures, based on COVIDNet-CXR3-A, COVIDNet-CXR4-A, AlexNet, VGG-11 and ResNet-50 for the classification of these different CXR representations separately. Finally, we summarize the results, and offer perspectives into the future of automated image classification using deep learning. The full pipeline of our proposed experiments is illustrated in Fig. 2, which emphasizes the creation of different CNN models and evaluation using Grad-CAM to highlight what regions the neural networks consider important for classification in each of these cases.

## Related work

Clinical imaging such as chest X-rays holds the promise to democratize access to early screening, particularly for symptomatic staging and treatment arrangements. That is because it enables rapid triage in resource-constrained and/or overwhelmed areas, improving availability and accessibility while using equipment broadly available at medical facilities throughout the world. As immediate treatment and care continue to challenge the healthcare systems worldwide, screening rules became essential to quickly isolate potential infected patients in order to mitigate the spread of the virus. In response, a community of investigators coalesced around using public datasets of CXR images, some also including metadata with respiratory conditions.

There have been various studies published in the area of deep learning for COVID-19 detection. Oh et. al.^18^ use patches of CXR images as input to a neural network in order to improve classification results for a small COVID-19 dataset. That paper uses data normalization by type casting images to float32, followed by histogram equalization, then gamma correction, and then image resizing to 256 × 256 pixels. After image preprocessing, the network is trained with random patches from the lungs to detect COVID-19. For inference, a hundred random patches from the test set are used to evaluate the network performance, using the majority rule as the decision for the network, finally reporting accuracy of 91.9% in classifying CXR images. Apostolopoulos et al.^12^ evaluate the utility of transfer learning for classifying COVID-19 using CXR images by testing various neural network architectures (VGG-19, Mobilenet v2, Inception, Xception, and Inception ResNet v2), reporting an accuracy of up to 98.75% (VGG-19) based on a 2-class approach. Vaid et al.^19^ evaluate the performance of a modified pretrained VGG-19 network for classifying COVID-19 vs. normal CXR scans, reporting 96.3% accuracy. Hussain et al.^20^ propose a novel architecture for COVID-19 classification named Corodet, reporting accuracy of 99%, 94.2%, 91.2% for the 2-, 3-, and 4-class detection problems respectively. Rajaraman et. al.^21^ propose a method for weakly labeled data augmentation as a means to expand the training dataset and improve performance results. Nayak et al.^22^ evaluate the performance of pre-trained Resnet-50, ResNet-34, Inception-V3, SqueezeNet, MobileNet-V2, GoogleNet, AlexNet, VGG-16 on the task of classification of COVID-19 from normal CXR images, and ResNet-34 was reported to have the highest accuracy of 98.33%. Jain et. al^23^ compare the performance of Inception-V3, Xception, and ResNeXt for the COVID-19 classification task, finding that the Xception model offers the highest performance at 97.97% accuracy. A commonality among these experiments is the use of multiple CXR data sources, with the bulk of COVID-19 images coming from a separate data source. After considering an assortment of different image preprocessing steps and augmentation strategies, most of the approaches report better results using residual neural nets.

Nishio et al.^10^ compare the performance of several pre-trained models (VGG16, Resnet-50, MobileNet, DenseNet-121, EfficientNet) utilizing data augmentation for training on the task of classification of COVID-19 Pneumonia, Non-COVID Pneumonia, and normal CXR images, reporting the best accuracy from VGG-16 as 83.68%. Bressem et al.^9^ evaluate the performance of 16 different networks based on ResNet, DenseNet, VGG, SqueezeNet, Inception-V4 and AlexNet for the task of COVID-19, pneumonia, and normal classification using a public COVID-19 image data collection, as well as the task of classifying cardiomegaly, edema, consolidation, atelectasis and pleural effusion using the CheXpert Dataset. They observed AUROCS between 0.83 and 0.89 for the CheXpert classification task, and AUROCS between 0.983 and 0.998 for the COVID-19 Image Collection dataset Classification task. Stubblefield et al.^24^ explore the utility of using deep neural networks used as feature extractors for classical networks to be applied on smaller datasets; they utilize a deep neural network trained on the CheXpert dataset for image feature extraction and XGBoost as the final algorithm for performing the classification task on their small dataset aimed at classification of cardiac vs infectious etiologies of ARDS. They also evaluated the performance of inclusion of clinical features into the final classifier for the predictive model. For the infection label they achieved 67.5% accuracy, and for the cardiac labeling task reached 74.5%. Sahlol et al.^25^ evaluate the performance of various neural networks including Inception V3 on the COVID-19 classification task, and additionally show that performance can be improved when using the Marine Predators Algorithm for feature selection from Inception V3 while minimizing network size, reaching accuracy of 0.9877 and 0.9968 for two separate datasets. Despite increased efforts toward standardizing data sources and metrics for comparison, these articles also considered different data sources to create a suitable CXR image set for all the classes to be well-represented.

After the public release of COVIDx dataset^13^, containing hundreds of COVID-19 CXR images, more than five hundred research works leveraged variants of that workflow and/or datasets, many proposing CNNs to evaluate computer vision tasks such as lung segmentation, and classification of COVID-19 cases^26–28^. One of the most prominent CNNs was COVID-Net^13^, which was proposed as a prediction engine to gain insights into important factors associated with COVID-19 cases imaged using CXR. Such work also attempted to verify that the decision-making is based on relevant information from the images. While representing an invaluable step forward in COVID-19 research, particularly regarding reproducibility, the authors state that they do not mean it is a production-ready solution. Our work investigate to which extent previous work on CXR could be leveraged as part of software that can improve prescreening. In order to address critical issues in using deep learning, we created a set of protocols and strategies to validate inference models based on CNNs using such CXR data sources, and audit if the learned models used radiological signatures rather than random artifacts, such as bright spots outside of the body, electrodes, and/or markup symbols.

## Materials and methods

This section describes the different CXR image datasets we use to accomplish two main tasks: (a) CXR segmentation into lung area and non-lung; (b) CXR classification into normal, pneumonia and COVID-19.

### Lung segmentation with U-Net

#### Segmentation data

This paper investigates the dataset curated and made publicly available by Tang et al.^16^, in which they utilized the MUNIT method^29^ to generate an augmented lung segmentation dataset using image-to-image translation. For training, we use the “Augmentation” dataset that contains 2400 images and corresponding masks and for testing we use their “NIH” dataset, which contains additional 100 images and masks. We perform additional testing using 206 images and masks available in the Cohen dataset as well.

#### Segmentation method

For the purposes of the segmentation task, we use an implementation of the U-Net^30^, a deep neural network architecture that has shown to have strong performance in various biological image segmentation tasks^31–34^. The configurations for the proposed U-net are: batch size of 1, and a learning rate of .0001, and trained for 50 epochs/until convergence. This U-Net model is then used to segment the training images in the COVIDx5 dataset in order to remove or isolate the ROI’s from the images.

### CXR classification

#### Classification data

As discussed in^13^, the COVIDx dataset combines 5 different publicly available data repositories, containing images that can be classified into normal (no pneumonia), non-COVID-19 pneumonia, and COVID-19 patients. These repositories are: (1) COVID-19 Image Data Collection, (2) the COVID-19 Chest X -ray Dataset Initiative, (3) the ActualMed COVID-19 Chest X-ray Dataset Initiative, (4) RSNA Pneumonia Detection Challenge dataset, which leveraged CXR data from a non-COVID-19 work, and (5) the COVID-19 radiography database.

While undoubtedly a relevant contribution as a publicly available data repository, COVIDx also comes with caveats, for example, the distribution of patient images across the different infection types. As illustrated in Table 1, the number of images is highly skewed toward non-COVID-19 cases. To exacerbate this issue, COVID-19 images arose from one data source while images from other cases come from different origins. Therefore, these images are potentially influenced by features derived from acquiring data from different instruments and their respective artifacts.

**Table 1:** COVIDx V5 chest radiography images distribution.

Partition	Normal	Pneumonia	COVID-19	Total
Train	7966	5475	517	13,958
Test	885	594	100	1579

#### ROI Hide-and-Seek protocol for data representation

Using the segmentation results, we modify the COVIDx5 dataset in four different ways. We first use the segmentation to isolate the lungs, and call this version of the dataset “lungs isolated”. We then do the inverse operation and remove the lungs using the segmentation and name this dataset “lungs removed”. In a third dataset called “lungs framed”, we also use the segmentation to form bounding boxes around the lung and remove everything outside of the boxes. We also perform a similar inverse operation and name this dataset “lungs boxed out”. These will be treated as separate datasets used for training and testing to evaluate whether the networks are able to learn when the image data includes or excludes the presumed ROI. Examples of these datasets are shown in Fig. 1. In order to avoid artifacts from the lung-removal operator, for example, the remainders of lung borders or lung shape that might bias the model, we have also included the lung-boxed-out operator. The expectation is that this set of protocols will enable clearer interpretations regarding accuracy metrics and presence of the ROI of interest in the input.

#### Data preprocessing and augmentation

Wang et al.^13^ performs various data prepossessing and augmentation functions for training and testing purposes. They first crop the top 8% of the images in order to remove metadata. Their provided training scripts use the following augmentations: random ratio resizing, $\pm 10\%$ degree random rotation, $\pm 10\%$ horizontal and vertical translation, $\pm 15\%$ zoom, $\pm 10\%$ intensity shifts, and horizontal flipping. For the purposes of retraining their provided models from scratch using their own scripts on the raw and modified COVIDx5 datasets, we use all of these augmentations. However, for our own experiments training ResNet-50, VGG-11, and Alexnet, we consider each of these augmentations with the exception of horizontal flipping, which is anatomically unrealistic since each image in the dataset is an anterior X-ray image.

#### Training

We created a modified version to the Tensorflow^36^ scripts provided by Wang et al.^13^ for training their provided models from scratch. For example, the derived scripts load information from the provided .meta files without the weights. Our scripts train the networks using a learning rate of 0.0002 and a batch size of 8. We train the COVID-Net CXR3A and 4A models for 10 epochs each. They also employ the softmax cross entropy loss function and a class weighting scheme of 1,1,4 for the normal, pneumonia and COVID-19 classes respectively. They also use data batch re-balancing to ensure that each batch is balanced in terms of the classes. Our modified version seeks to recreate the experiments as closely as possible of their work without having access to GenSynth^37^, as their model was built automatically from a pre-trained baseline model, which is not available.

For our own training experiments with ResNet-50, VGG-11, and AlexNet, we use the Pytorch deep learning framework^38^, considering models pre-trained with ImageNet. We train each network with a batch size of 32 and 0.0001 learning rate for 20 epochs, considering the cross entropy loss with class weights of 1, 1, 4 for normal, pneumonia and COVID-19 classes respectively. We also use a data sampler to ensure that the data batches are balanced across the three datasets as well.

In order to speed up our computations, we run these experiments on a high performance supercomputer called Cori, which is a Cray XC40 with a peak performance of about 30 petaflops, comprised of 2388 Intel Xeon “Haswell” processor nodes and 9688 Intel Xeon Phi “Knight’s Landing” (KNL) nodes, also with access to GPUs. These resources are available at the National Energy Research Scientific Computing facility (NERSC) at Lawrence Berkeley National Laboratory.

**Figure 3:**
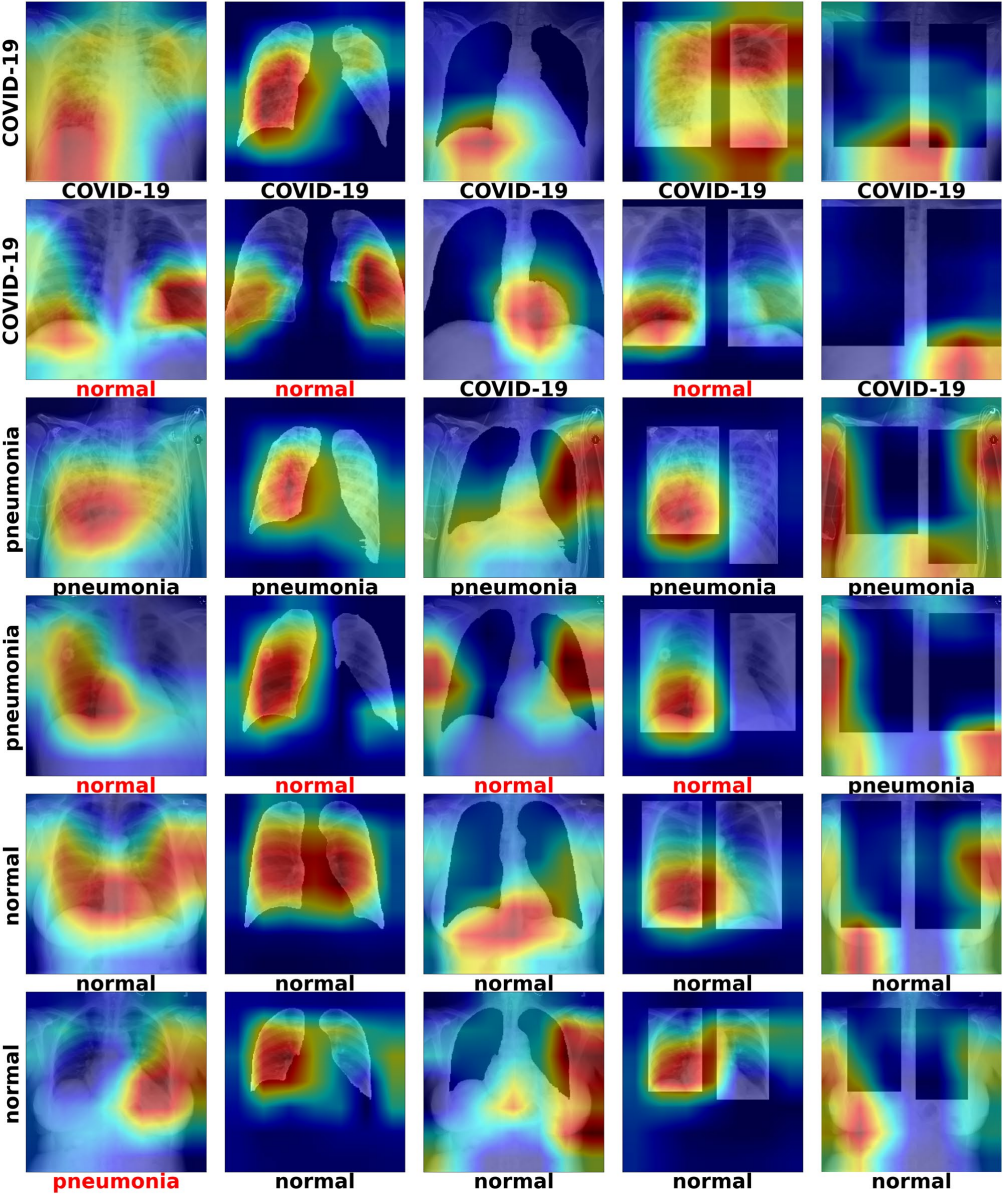
Grad-CAM of ResNet-50 models—the label on the left side of each row corresponds to the ground-truth label of that image (COVID-19, Pneumonia, Normal). Each image in each row corresponds to the Grad-CAM inference result on the different modified test sets (data, data_lungs_isolated, data_lungs_removed, data_lungs_framed, data_lungs_boxed_out). The label underneath the individual images corresponds to the classification label generated by the network corresponding to that modified version of the dataset.

Figure 3 illustrates results of running Grad-CAM on CXR images with examples labeled as normal, pneumonia and COVID-19.

## Results

Table 2 shows the performance metrics of our U-Net segmentation model on both the NIH test set and Cohen dataset. It is observed that we reach an F1 score of .95 and .918 for the “lung” class for the NIH and Cohen datasets respectively. This is satisfactory performance for our purposes of masking out or isolating the ROI in the COVIDx dataset. Figure  1 visualizes the sample image from the resulting datasets generated using the segmentation results. Each modified version of the COVIDx5, obtained with the ROI-Hide-and-Seek, will then be used to train and test each of the neural network models separately. As a result, this paper reports on the performance metrics when using COVID-Net CXR3-A as in Table 3, and also on a more recent version of that model, COVID-Net CXR4-A as in Table 4. In addition, we also check the classification performance of AlexNet as in Table 5, VGG-11 as in Table 6 and ResNet-50 as in Table 7. All of these tables show the test accuracy and class-wise performance metrics (sensitivity, positive predictive value) in different columns, with each row showing the results corresponding to each of the individual modified datasets, namely lungs_isolated, lungs_removed, lungs_framed, lungs_boxed_out.

**Table 2:** Accuracy metrics for lung segmentation from CXR based on U-net applied to two public datasets: NIH^35^ and Cohen^17^.

Metric	NIH dataset		Cohen dataset	
	Class 0	Class 1	Class 0	Class 1
Recall	0.981	0.968	0.971	0.919
Precision	0.991	0.934	0.971	0.923
Jaccard	0.973	0.906	0.945	0.854
F1	0.986	0.95	0.971	0.918
Accuracy	0.979	0.979	0.958	0.958

class 0 = background, and class 1 = foreground/lung area.

**Table 3:** Performance metrics using different data preparation strategies classified with COVIDNet-CXR3-A model: sens and pneumo stand for sensitivity and pneumonia, respectively.

Dataset	Accuracy	Sens normal	Sens pneumo	Sens covid	PPV normal	PPV pneumo	PPV covid
Data	0.91	0.95	0.91	0.87	**0.896**	0.867	**0.978**
Lungs_isolated	0.89	0.94	0.82	0.91	0.803	**0.943**	0.948
Lungs_removed	0.903	**0.96**	0.81	**0.94**	0.842	0.931	0.949
Lungs_framed	**0.917**	0.92	**0.92**	0.91	0.868	0.929	0.958
Lungs_boxed_out	0.847	0.84	0.82	0.88	0.8	0.812	0.936

Bold values correspond to best performance metric for dataset in the same row.

**Table 4:** Performance metrics using different data preparation strategies classified with COVIDNet-CXR4-A model.

Dataset	Accuracy	Sens normal	Sens pneumo	Sens covid	PPV normal	PPV pneumo	PPV covid
Data	**0.913**	**0.96**	**0.89**	0.89	0.857	0.937	0.957
Lungs_isolated	0.903	0.95	0.85	0.91	0.848	**0.966**	0.91
Lungs_removed	**0.913**	0.95	0.85	**0.94**	0.856	0.924	**0.969**
Lungs_framed	0.897	0.91	**0.89**	0.89	**0.858**	0.899	0.937
Lungs_boxed_out	0.863	0.84	0.83	0.92	0.832	0.83	0.929

Bold values correspond to best performance metric for dataset in the same row.

**Table 5:** Performance metrics using different data preparation strategies classified with pre-trained AlexNet model.

Dataset	Accuracy	Sens normal	Sens pneumo	Sens covid	PPV normal	PPV pneumo	PPV covid
Data	**0.917**	**0.95**	0.89	**0.91**	**0.88**	**0.927**	0.948
Lungs_isolated	0.887	0.9	0.87	0.89	0.857	0.906	0.899
Lungs_removed	0.873	0.87	**0.9**	0.85	0.837	0.841	0.955
Lungs_framed	0.91	0.92	**0.9**	**0.91**	0.893	0.882	**0.958**
Lungs_boxed_out	0.817	0.84	0.76	0.85	0.785	0.817	0.85

Bold values correspond to best performance metric for dataset in the same row.

**Table 6:** Performance metrics using different data preparation strategies classified with pre-trained VGG-11 model.

Dataset	Accuracy	Sens normal	Sens pneumo	Sens covid	PPV normal	PPV pneumo	PPV covid
Data	**093**	0.94	**0.92**	0.93	**0.913**	0.92	**0.959**
Lungs_isolated	0.92	**0.99**	0.89	0.88	0.868	**0.957**	0.946
Lungs_removed	0.91	0.91	0.9	0.92	0.892	0.9	0.939
Lungs_framed	0.917	0.92	0.89	**0.94**	0.911	0.918	0.922
Lungs_boxed_out	0.893	0.89	0.88	0.91	0.864	0.871	0.948

Bold values correspond to best performance metric for dataset in the same row.

**Table 7:** Performance metrics using different data preparation strategies classified with pre-trained ResNet-50 model.

Dataset	Accuracy	Sens normal	Sens pneumo	Sens covid	PPV normal	PPV pneumo	PPV covid
Data	**0.933**	0.95	**0.93**	0.92	0.905	0.921	**0.979**
Lungs_isolated	0.917	0.96	0.89	0.9	0.873	**0.957**	0.928
Lungs_removed	0.927	0.93	0.92	**0.93**	**0.93**	0.902	0.949
Lungs_framed	0.92	**0.99**	0.87	0.9	0.884	0.956	0.928
Lungs_boxed_out	0.897	0.94	0.86	0.89	0.832	0.925	0.947

Bold values correspond to best performance metric for dataset in the same row.

Notice that each row corresponds to the test results of training each architecture from scratch on that specific modified version of the COVIDx dataset. It can be observed that the networks each have relatively high accuracy, even when trained and tested on datasets where the ROI lung regions are completely removed via segmentation (lungs_isolated) and bounding boxes (lungs_boxed_out), reaching up to accuracy of .927 and .897 for each of these modified datasets respectively (see Table 7). Additionally, the test accuracy reached using each of these datasets never falls below .873 and .817 respectively (see Table 5). Most importantly, when compared to the accuracy using the original dataset, we observe that removing the ROI only results in a loss of accuracy between 0% and 4.4% for the lungs_removed dataset 3.6% and 10% for the lungs_boxed_out date-set.

It can also be observed that the class-wise performance metrics (sensitivity, positive predictive value) also remain relatively high across all different modifications of the dataset as well, implying that each class is still predicted well even when the ROI is removed.

### Architecture comparisons

Despite considering architectures proposed in Wang et. al^13^, we developed several adaptations and improvements to circumvent the lack of reproducibility in their original proposal as well as introduce essential methods for scrutinizing classification schemes, which is the case of our proposed ROI Hide-and-Seek protocol. The original COVID-Net model is described in their paper, but that, along with the other models’ architecture formations, are developed via the GenSynth framework. Unfortunately, that software is proprietary, therefore it is very difficult to reproduce the results and development of the network. This also makes it unclear how the new network models were created following the original paper, such as the CXR4-A model.

### Findings and interpretation

Tables 3, 4, 5, 6, 7 shows that well-known artificial neural network architectures led to similar representational capacity and accuracy when compared to the COVID-Net, despite the fact that the latter was created via a human-machine collaborative design strategy.

The results of this study demonstrate that each of the neural network architectures used for classifying lung CXR images is able to predict whether a lung scan belongs to a COVID-19 patient with high accuracy even when the dataset is modified to remove the lungs from the image. When we remove significant parts of the image that are within the main ROI by segmenting the lungs, we see relatively little change in accuracy. This calls into question what features the neural network actually extract when optimizing its parameters for the classification problem. ResNet-50 in particular is able to reach up to .897 (see Table 7) accuracy even for the most aggressive ROI removal case of lungs_boxed_out. This could imply that the features that the neural network extracts and uses for classification in fact exist outside of the lung as well. In the case of the CXR3 and CXR4 models, it can additionally be observed that the network performs better when the lung is removed from the image vs. when it is isolated. This could imply that significant amounts of information that contribute to classification in fact exist outside the lung regions, which is unexpected of a lung-based illness.

Instead of using GSInquire^13^ to highlight critical factors, we use Grad-CAM^39^ to provide explainable reasons for the classifications results and further interpretation. Figure  3 shows the Grad-CAM results using test data of the ResNet-50 models trained on the different datasets. We show specific cases where all network classifications agree (bottom labels) and their respective ground truth (left hand side labels).

For datasets where lungs are visible in the images, the lungs are typically highlighted as the important feature. However, in datasets where the lungs are removed, the Grad-CAMs shift towards other regions such as the stomach and arms. These results are difficult to interpret, as it seems that the important features extracted by the neural network are entirely dependent on whatever information is presented to it, regardless of whether it makes sense from a domain knowledge standpoint. One of the main assertions of Wang et. al^13^ paper is that the Grad-CAMs can be used to interpret and isolate unhealthy parts of the lungs; however, these observations seem to suggest that what they highlight may be arbitrary.

## Discussion

The classification of lung CXR for COVID-19 prescreening continues to be controversial, and some additional issues have been highlighted in this paper. Initially, we expected that the lung segmentation would enable data reduction and increase accuracy of the networks, or would reduce convergence time during network training since in theory this would allow the network to focus on the ROI. However, what we found out was that ROI isolation led to decreased accuracy when using any of the deep learning models for classification.

For example, we see a decline in accuracy of ResNet-50 of about 2% when isolating the lungs with the segmentation, and about 1.3% when using a bounding box to isolate the lungs. In order to better understand how much of the information outside of the lungs played a role in the classification results, we performed the same exact training but with the lungs removed from the images. What we found out was that when the lungs are removed, the network’s accuracy barely changes, and in some cases, actually outperforms the network with the lungs isolated. This might suggest that there is contextual information outside of the lungs that may be contributing to the classification results in the original/unmodified dataset. By running Grad-CAM, we highlight the different possible regions that might be affecting the classification results, which are potentially information that is related to the data sources instead of being related to COVID-19.

Overall, we observed that the main issues of current approaches proposed to automate COVID-19 screening and/or diagnosis of CXR are: (a) the lack of clarity about which features are being detected when using deep learning algorithms, which can bring serious consequences to image classification, such as identification of image landmarks that are clinically irrelevant to COVID-19; (b) the lack of more systematic ways of testing classification strategies that prevent skewed conclusions regarding the use of convolutional neural networks; and (c) the need for more clear protocols for normalization of data coming from different sources, which are key to distinguish correlations associated to a particular data source as opposed to clinical usefulness of CXR image classification for respiratory illnesses. These issues are important in testing and and comparing deep learning algorithms for CXR classification.

## Data Availability

The datasets generated during and/or analyzed during the current investigation are available in the github repository: https://github.com/Electro1111/COVID_19_CXR_CLASSIFICATION.
